# Extracellular vesicles from human umbilical cord blood plasma modulate interleukin-2 signaling of T cells to ameliorate experimental autoimmune encephalomyelitis

**DOI:** 10.7150/thno.42742

**Published:** 2020-04-06

**Authors:** Sueon Kim, Ji-young Maeng, Seung-Joo Hyun, Hyun-Jung Sohn, Su-Yeon Kim, Cheol-Hwa Hong, Tai-Gyu Kim

**Affiliations:** 1 Department of Microbiology, College of Medicine, The Catholic University of Korea, Seoul, Republic of Korea;; 2 Department of Biomedicine and Health Sciences, College of Medicine, The Catholic University of Korea, Seoul, Republic of Korea;; 3 Catholic Hematopoietic Stem Cell Bank, College of Medicine, The Catholic University of Korea, Seoul, Republic of Korea.

**Keywords:** interleukin-2 (IL-2) signaling, matrix metalloproteinase-9 (MMP-9), regulatory T cell (Treg), umbilical cord blood plasma-derived extracellular vesicles (CBP EVs), experimental autoimmune encephalomyelitis (EAE)

## Abstract

Human umbilical cord blood (UCB) cell-derived extracellular vesicles (EV) reportedly play immunosuppressive roles; however, UCB plasma-derived extracellular vesicles (CBP EVs) remain poorly studied. We examined the immunosuppressive potential of CBP EVs compared to that of adult blood plasma-derived extracellular vesicles (ABP EVs) *in vitro* and constructed an experimental autoimmune encephalomyelitis (EAE) model.

**Methods**: CBP EVs were isolated by ultracentrifugation and their proteomic profiling was performed using the high-resolution liquid chromatography with tandem mass spectrometry. Human T lymphocytes or mouse splenocytes labeled with carboxyfluorescein succinimidyl ester were incubated with CBP EV to measure the immunosuppressive function of CBP EV. The effect on T-cell polarization was analyzed by flow cytometry and enzyme-linked immunospot assay. The matrix metalloproteinase (MMP) function in CBP EV was specifically inhibited using a chemical inhibitor. The efficacy of CBP EVs in the EAE mouse model was determined by scoring the symptoms and analyzing cell phenotype and cytokines using mouse splenocytes. We generated genetically engineered artificial EVs using HLA/MIC-null HEK293T (H1ME-5) cell line to characterize the immunosuppressive effect of CBP EV.

**Results**: CBP EVs primarily inhibited the proliferation of T cells by reducing the production of IL-2. Specifically, CBP EV-derived matrix metallopeptidase cleaved the IL-2 receptor α (CD25) on the surface of activated T cells, consequently downregulating IL-2 signaling in response to IL-2R engagement. Although the inhibition of MMP activity in CBP EVs abrogated CD25 cleavage and restored IL-2 production in activated T cells, the immunosuppressive response was not fully recovered. Thus, we further analyzed changes in immunosuppressive cells such as regulatory T cells and bone marrow-derived suppressor cells by CBP EV. Further, GAL-3, GAL-7, S100-A7, MMP-9, MMP-8, HSP-72, and PIP were highly enriched in CBP EV-mimics in which they served as pivotal mediators of CBP EV-induced immunosuppressive effects. Therefore, we generated genetically engineered GAL-3, GAL-7, S100-A7, MMP-9, MMP-8, HSP-72, and PIP-EVs using HLA/MIC-null HEK293T cells to characterize the immunosuppressive effect of these molecules. Among these, MMP-9 and HSP-72-enriched EVs showed the most significant T cell immunosuppression.

**Conclusion**: CBP EVs inhibited T cell proliferation and EAE development by modulating IL-2 signaling and immunosuppressive cell fate. CBP EVs contain critical components for immunosuppression and that CBP EV mimics, specifically those expressing MMP-9 and HSP-72, may offer a novel promising strategy for the treatment of various autoimmune diseases.

## Introduction

Human umbilical cord blood (UCB) has been used as a source of hematopoietic stem cells for transplantation. However, UCB also contains numerous other cells, such as immune cells, mesenchymal stromal cells, and endothelial progenitor cells 1, 2. The immune tolerance of a fetus, through the maternal immune system, plays a crucial role in the maintenance of pregnancy 3. Immunological interactions between the fetus and the mother represent a bi-directional communication system controlled by the presentation of fetal antigens and/or the recognition of and response to these antigens by the maternal immune system 4. UCB-derived cells have specific immunomodulatory properties that contribute to maintaining pregnancy 5, 6. Moreover, UCB is also a rich source of immunosuppressive cells, such as regulatory T cells (Tregs) 7, and myeloid-derived suppressor cells (MDSCs) 8.

EVs are small membrane vesicles that harbor unique subsets of proteins with roles that reflect their source and environment 9-12. As UCB-derived cells can be selected and expanded ex vivo 7, 13-15, EVs derived from these cells can be efficiently isolated from cell culture supernatants. EVs that are released from UCB-derived cells may have immunosuppressive effects 16, 17, as they can induce the differentiation of circulating immunosuppressive cells, such as Tregs and MDSCs 18-21. While plasma and serum biofluids are the main sources, other biofluids may represent valuable sources of EVs 22, 23. However, despite the progress in research on UCB-derived cells and EVs, human UCB plasma-derived extracellular vesicles (CBP EVs) remain poorly studied. Proteomic analysis of CBP EVs has revealed that exosomal proteins are associated with T cell proliferation, differentiation, negative regulation, membrane permeability, wound healing, arginase activity, and enzyme modulatory activity 24. Of the plethora of functions exerted by CBP EVs, only their wound healing function has been studied in detail 25. However, no studies have been reported on the immunosuppressive function of CBP EVs.

In addition to UCB-derived cells, umbilical cord blood plasma (CBP) plays a role in suppressing T cell proliferation by inhibiting interleukin-2 (IL-2) signaling, through its various immunomodulatory factors 25-27. IL-2 and its receptor, IL-2R, play a role in supporting the proliferation and survival of T cells and differentiation of naive T cells. The CD25 is an essential component of IL-2R 28. Moreover, the surface expression of CD25 on CD4^+^ T cells and the level of IL-2 in activated T cells are significantly reduced by matrix metalloproteinase-9 (MMP-9) 29. The inhibition of MMP activity by chemical inhibitors restores IL-2 production and partially rescues T cell proliferation 29. Because CBP immunoregulatory factors, as well as MMP-9, are found in CBP EVs 30, these EVs are expected to harbor substantial immunosuppressive potential.

It is an exciting scientific challenge to characterize better and further modify the composition of these vesicles, to engineer EVs to be implemented as a delivery system for therapeutic purposes 10, 31, 32. The CRISPR/Cas9 system is a high-throughput multiplex genome editing tool 33, 34. It can be used to delete HLA class I and MICA/B genes in the human embryonic kidney (HEK) 293T cells by inducing multiple, large gene deletions 35. This HLA/MIC null-293T (H1ME-5) cell line could be applied as a CBP EV mimic by transferring CBP EV-associated genes, such as GAL-3, GAL-7, S100-A7, MMP-9, MMP-8**,** HSP-72, and PIP. Our study suggests that EVs could inhibit the proliferation of T cells via these immune modulation-associated proteins. We found that MMP-9 was a pivotal mediator among the various proteins involved in the CBP EV-induced regulation of T cells.

To the best of our knowledge, this study is the first to verify the immunosuppressive role of CBP EVs -related proteins. We also aimed to investigate the immunosuppressive functions of CBP EVs as compared to those of adult blood plasma-derived extracellular vesicles (ABP EVs), both *in vitro* and in a mouse model of experimental autoimmune encephalomyelitis (EAE).

## Methods

### Human samples

Human peripheral blood mononuclear cells (PBMCs) and human UCB were provided by the Catholic Hematopoietic Stem Cell Bank after written informed consent was provided by healthy donors or normal full-term pregnant women. The study involving human subjects was carried out in accordance with the recommendations of the Declaration of Helsinki. The protocol was approved by the institutional review board of the College of Medicine, Catholic University of Korea, Seoul, Republic of Korea (permit No. MC18SESI0003, MC16SISI0084). All subjects gave written informed consent for sample donation in accordance with the Declaration of Helsinki.

### Mice

C57BL/6 mice were purchased from OrientBio, Inc. (Seoul, Korea) and maintained under specific pathogen-free conditions according to the guidelines of the Institute of Laboratory Animal Resources of the Catholic University of Korea. All animal experiments were approved by the Institutional Animal Care and Use Committee of the Catholic University of Korea. All animal experiments were performed according to the investigator's protocol approved in advance by the Institutional Animal Care and Use Committee, College of Medicine, Catholic University of Korea (permit No. CUMC-2017-0273-05).

### EVs isolation

Human adult blood plasma (ABP) and CBP EVs were taken immediately after delivery, from the Catholic Hematopoietic Stem Cell Bank and were freshly isolated using the umbilical cord blood, which was below the reference weight according to the umbilical cord blood management regulations. CBP, ABP, and the culture supernatants of HEK293T were first centrifuged at 400 × g for 5 min and then at 2,000 × g for 10 min, followed by a membrane filtration step using a 0.22 μm polyvinylidene fluoride membrane (Nalgene™, Rochester, NY) to remove the cells, cell debris, and microvesicles from the sample. The EVs were then separated using ultracentrifugation. The protein yield of each CBP or ABP EV sample was determined by a NanoDrop spectrophotometer (Thermo Scientific, San Diego, CA) set at an absorbance of 280 nm. Umbilical CBP was ultra-centrifuged at 100,000 × *g* for 2 h, and the CBP pellet was used for comparative analysis. As a control, adult blood plasma was isolated and subjected to the same EV isolation procedure. All fractions were maintained at 4 °C and either used within 24 h for *in vitro* experiments or frozen at -80 °C. CBP EVs were obtained by continuously collecting CBP samples from a total of 10 healthy donors per batch. Character analysis of EVs were performed for each batch using the Exo-Check EV Antibody Array (System Biosciences, Palo Alto, CA) or PE-conjugated anti-human CD9 (e-Bioscience, San Diego, CA), anti-human CD63 (BD Biosciences, San Jose, CA), anti-human CD82 (Biolegend, San Diego, CA), or anti-human HSP70/HSP72 (Enzo Life Sciences, Farmingdale, NY) FACS antibodies. The EV Antibody Array Kit consists of a standard exosomal protein as a positive control and a blank as a negative control.

### EV size and particle number analysis

EVs obtained after differential centrifugation were suspended in PBS. Ten micrograms of EVs suspension were loaded onto formvar carbon-coated 200 mesh copper grids for 10 min at room temperature (25 °C). The excessive fluid was slightly drained with filter paper. Adsorbed EVs were negatively stained with 1% phosphotungstic acid for 5 min. Finally, the air-dried EV-containing grids were observed by a transmission electron microscope (JEM-1010, Japan) operating at 100 kV. The size distributions of the nanoparticles were determined by dynamic light scattering (DLS) using the Malvern Zetasizer Nano ZS instrument. The EV concentration, defined by the number of nanoparticles, and the more precise sizes of the EV were determined by nanoparticle tracking analysis (NTA) carried out using the NanoSight NS 300 system. Particle count and size of EV samples were measured by nanoparticle tracking analysis (NTA) using a Nanosight NS300 system (Malvern Instruments Ltd). The system was calibrated and cleaned between samples with ultrapure MilliQ water. Each sample was diluted 1:200 to 1:4000 in DPBS (Gibco) to reach a concentration within the recommended measurement range (1-10 × 108 particles/ mL), loaded into 1 mL syringes and infused into the NS300 system using a syringe pump (Harvard Apparatus, Cat# 98-4730). Each measurement was recorded in a set of three videos of 30 s each with a 5 s delay between recordings under the same setting (Capture - screen gain 1.0, camera level 10; Process - screen gain 10.0, detection threshold 10), and the data were analyzed using NTA software version 3.0.

### Proteomic Analysis

Proteomic profiles of exosomes were analyzed by The Exosome Proteomics Service from System Biosciences (Mountain View, CA, USA).

### Transfection and cell sorting

HEK 293T cells were seeded at 2 × 10^6^ cells/10 mL in antibiotic-free Dulbecco's modified Eagle's medium (Lonza). Twenty-four hours later, a mixture of seven individual all-in-one plasmids specific for each of the seven targets were transfected into 293T cells using Lipofectamine reagent (Invitrogen, Carlsbad, CA). At 48 h after transfection, cells were analyzed by flow cytometry. At 6 days after transfection, cells positive for GAL-3, GAL-7, S100-A7, MMP-9, MMP-8, HSP-72, and PIP were sorted using Moflo XDP Cell Sorter (Beckman), and clones were established.

### Proliferation assay

Human PBMCs isolated from the blood of healthy donors 20-30 years old and Mouse splenocytes isolated from naive or EAE disease induced C57BL/6 mice. PBMCs or splenocytes (5 × 10^5^) in culture medium were seeded into 96-well tissue culture plates with anti-CD3/CD28 DYNABEAD (Invitrogen, Oslo, Norway). CBP EVs or ABP EVs were added into appropriate wells simultaneously. Cell proliferation was measured by 5,6-carboxyfluorescein diacetate-succinimidyl ester (CFSE) staining followed by flow cytometry. Briefly, 5 × 10^5^ splenocytes or PBMCs were incubated with 5 μM of CFSE (Molecular Probes, Eugene, OR) in 1 mL of phosphate-buffered saline for 10 min at 37 °C and were washed twice with ice-cold 10% fetal bovine serum-containing culture medium. The stained cells were stimulated with DYNABEAD in the presence of CBP EVs or ABP EVs, as described above. After incubation for the indicated time, the cells were acquired using FACSCanto (Becton Dickinson Biosciences, Heidelberg, Germany), and the data were analyzed using ModFit LT 4.0 software (Verity Software House, Topsham, ME). At least three independent experiments were performed for verifying the immunosuppressive effects of CBP EVs from 10 healthy donors on activated PBMCs or splenocytes.

### Apoptosis assay and cell cycle analysis

Apoptotic cells were stained with Annexin V and analyzed according to the manufacturer's instructions. Briefly, 1 × 10^6^ cells were resuspended in 1 mL of Annexin-binding buffer. Subsequently, 5 μL of working solution comprising APC-Annexin V or FITC-Annexin V (BD Biosciences, San Jose, CA) and 5 μL of working solution of 7AAD (Catalog #551076; BD Biosciences) were added to 5 × 10^5^ cells in 100 μL, and the mixture was incubated at 37 °C in 5% CO_2_ for 15 min. The assay data were analyzed using FlowJo v10. PBMCs were stimulated with DYNABEAD and incubated in a 96‐well flat‐bottomed plate for 12-48 h. The cells were harvested and fixed in 70% ethanol, and the cell pellet was stained with propidium iodide (PI; Sigma) solution supplemented with RNase A in PBS. The cell suspension was incubated in the dark at room temperature for 30 min. The DNA content was measured with FACS Canto (Becton Dickinson) and analyzed using ModFIT LT 4.0 software to determine the sub‐G1, G1, S, and G2 phases of the cell cycle.

### Protease inhibition

For inhibiting protease activity, 10 μg/mL of the MMP inhibitor GM6001 (Calbiochem, San Diego, CA) was added to CBP EVs and incubated for 2 h at 21 °C. GM6001-pretreated CBP EVs were used for further experiments as indicated.

### ELISPOT assay

The enzyme-linked immunospot (ELISPOT) assay was performed according to the manufacturer's protocol (BD Biosciences). Briefly, monoclonal antibodies specific for human IL-2 (Catalog #551076; BD Biosciences), interferon (IFN)-γ (Catalog #551083; BD Biosciences), and IL-17 (Catalog #SEL421; R&D Systems) were coated onto a microplate (Millipore, Billerica, MA). For *in vitro* experiments, cells cultured for 6 days were washed three times in medium, resuspended in 1 mL of medium, and incubated overnight, prior to the ELISPOT assay. For *ex vivo* experiments, myelin oligodendrocyte glycoprotein (MOG)33-55 was added to 96-well microplates at a concentration of 1 × 10^6^ cells/well in medium. The microplates were incubated for 20 h at 37 °C in a CO_2_ incubator. The microplates were then washed four times with wash buffer. The wells were then filled with 100 μL/well of biotinylated polyclonal anti-mouse IL-2, IFN-γ, and IL-17. The plates were incubated for 2 h at 21 °C, washed with buffer, and then incubated for 1 h at ambient temperature in the presence of 100 μL/well streptavidin-horseradish peroxidase. The unbound enzyme was subsequently removed by washing, and then the enzyme substrate solution was added. After the development of spots, the reaction was stopped by the addition of distilled water. The plates were inverted and allowed to dry overnight away from light. The number of spots corresponding to IL-2-, IFN-γ-, and IL-17-secreting cells was determined using an automatic AID-ELISPOT reader (AID Diagnostika GmbH, Strassberg, Germany).

### Assessment of Th1, Th2, and Th17 cytokines using the Cytometric Bead Array (CBA) Human and Mouse Th1/Th2/Th17 Cytokine Kit

The Human and Mouse Cytometric Bead Array (CBA) Th1/Th2/Th17 Cytokine Kit was purchased from BD Biosciences (Catalog #560484 and #560485). Fifty microliters of assay beads, 50 µL of detection reagent, and 50 µL of the studied sample or standard were added consecutively to each sample tube and incubated for 3 h at 21 °C in dark. The samples were then washed with 1 mL of wash buffer and centrifuged at 500 × *g* for 5 min. After discarding the supernatant, the pellet was resuspended in 300 µL of buffer and analyzed on the same day by flow cytometry.

### Induction of EAE and histological Analysis

Encephalomyelitis was induced in mice (n = 5 per condition) using the MOG35-55 peptide in complete Freund's adjuvant (CFA) with pertussis toxin (PTx) as described previously 36. Following the appearance of EAE symptoms, the mice were scored daily by two independent investigators (0, no sign of disease; 1, limp tail; 2, limp tail and partial hind limb weakness; 3, complete hind limb paralysis; 4, complete hind limb and partial front limb paralysis; 5, death), stratified, and assigned to separate test groups in order to obtain equally weighted average disease scores prior to experimental interventions. EVs were injected at 0 and 7 days (pre-onset stage of EAE) or every alternate day for 10 days (post-onset stage of EAE). EAE mice receiving PKH67-EVs were euthanized 24h after injection. Brain and spleen sections were observed under a Lionheart imaging system (BioTek Instruments, Winooski, VT). Images were processed using Gen5 software (BioTek Instruments). For histological analysis, routine histology methods were carried out to obtain morphological details of the brain tissue in EAE mice. Paraformaldehyde fixed tissues were embedded in paraffin, and serial sections (8 μm) were prepared. Sections were stained with the conventional hematoxylin and eosin (H and E) staining method. Digital images were taken using a ×20 objective. Brain sections were observed under a Lionheart imaging system (BioTek Instruments, Winooski, VT), and images were processed to measure infiltrated cell numbers and the inflammation area using Gen5 software (BioTek Instruments).

### Statistical analyses

The data were analyzed for statistical significance using Prism version 7.0 (GraphPad, San Diego, CA). Bartlett's test, one-way analysis of variance (ANOVA), or two-way ANOVA was performed to calculate the significance between groups. The results with *p* ≤ 0.05 were considered statistically significant.

## Results

### Molecular characteristics of EVs from CBP

**Figure 1:**
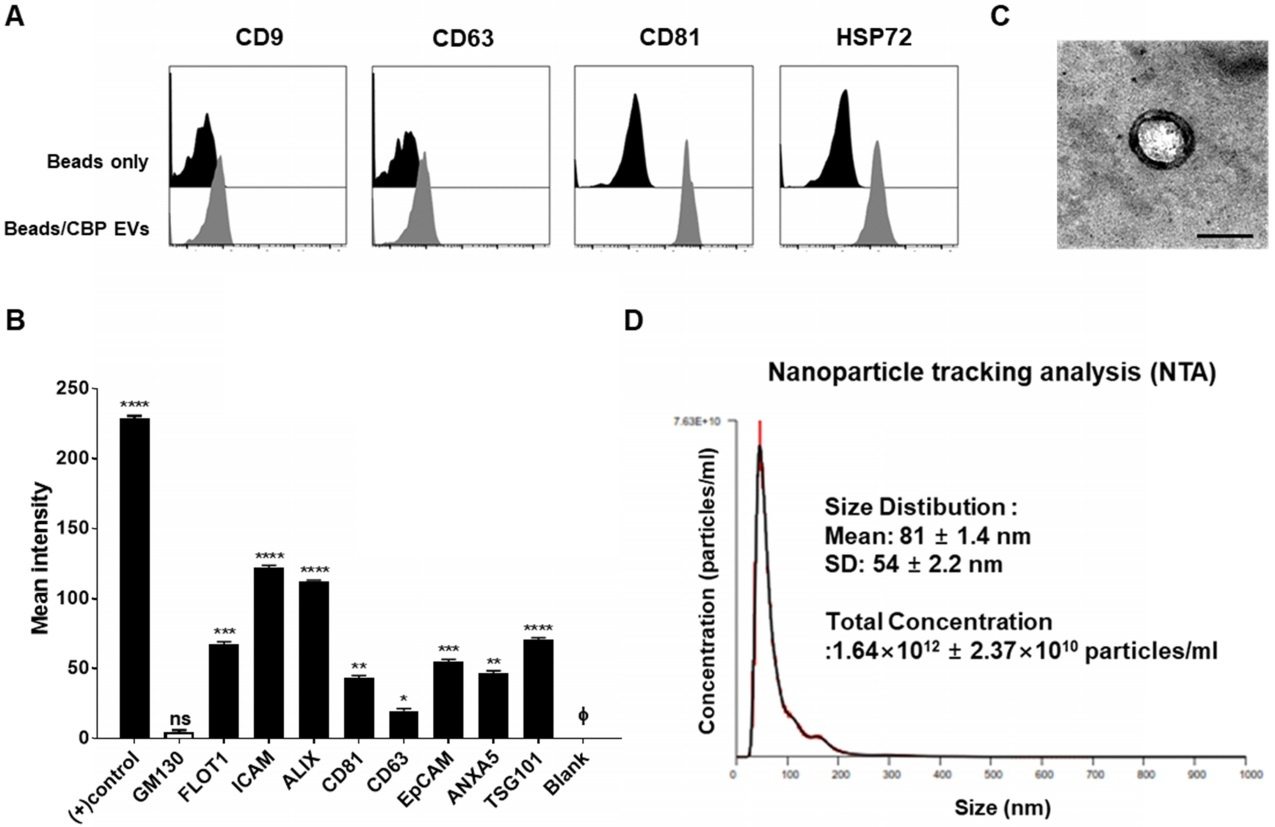
** Characterization of exosomal membrane vesicles purified from human umbilical CBP. (A)** CBP EVs bound to latex beads coated with individual EV-specific marker antibodies were analyzed by flow cytometry. Latex beads coated with complete exosomal preparations were included for comparison. The plots represent intensities derived from EV-specific antibodies (CD9, CD63, CD81 and HSP72) with the corresponding bead-only controls. **(B)** Dot-blot analysis of CBP EV lysates. The corresponding dots were evaluated using an EV antibody array kit. EV-specific antibody spots provided signals of varying intensities. The values shown are the means of values obtained in three independent experiments. Error bars = standard error of the mean (SEM). “Blank” indicates the negative control, also indicated by Φ, and GM130 represents cell debris. The mean intensity was analyzed using ImageJ software. **(C)** Particles with a lipid bilayer structure and the expected size (80-100 nm) were observed by TEM. Scale bars, 100 nm. **(D)** The CBP EVs are 81 ± 1.4 nm in size and 1.64×10^12^ ± 2.37×10^10^ particles/mL in number.

Using flow cytometry, we confirmed that CBP EVs contained the typical exosomal marker proteins CD9, CD63, CD81, and HSP72; however, these markers were absent on the bead-only control (Figure 1A). Moreover, the purity of these EVs was confirmed using an exosomal antibody array kit that included well-characterized exosomal protein markers such as CD81, ICAM, CD63, ALIX, TSG101, EpCAM, and FLOT-1, as well as the cis-Golgi marker GM130 for monitoring cellular contamination. Graphical representation of the results obtained from densitometric analysis using the EV antibody Array Kit with ImageJ software revealed the positive expression of specific exosomal markers. CBP EVs were highly positive for the exosomal protein markers FLOT1, ICAM, ALIX, CD81, TSG101, EpCAM, and ANXA5, whereas the expression of CD63 was low. The isolated EVs were free of cell debris and other contaminants, as demonstrated by negative staining for GM130 (Figure 1B). These results confirmed that the EVs isolated from CBP and CBP EVs showed similar results in different batches. EVs were also observed by transmission electron microscopy (TEM), and their size was roughly identical, ranging from of 80-100 nm and an average size of 81 ± 1.4 nm (Figure 1C-D). Moreover, the particle number of CBP EVs, as measured by NTA, was 1.64 × 10^12^ ± 2.37 × 10^10^ particles/mL (Figure 1D).

### CBP EVs, not ABP EVs, inhibit human T cell proliferative responses

**Figure 2:**
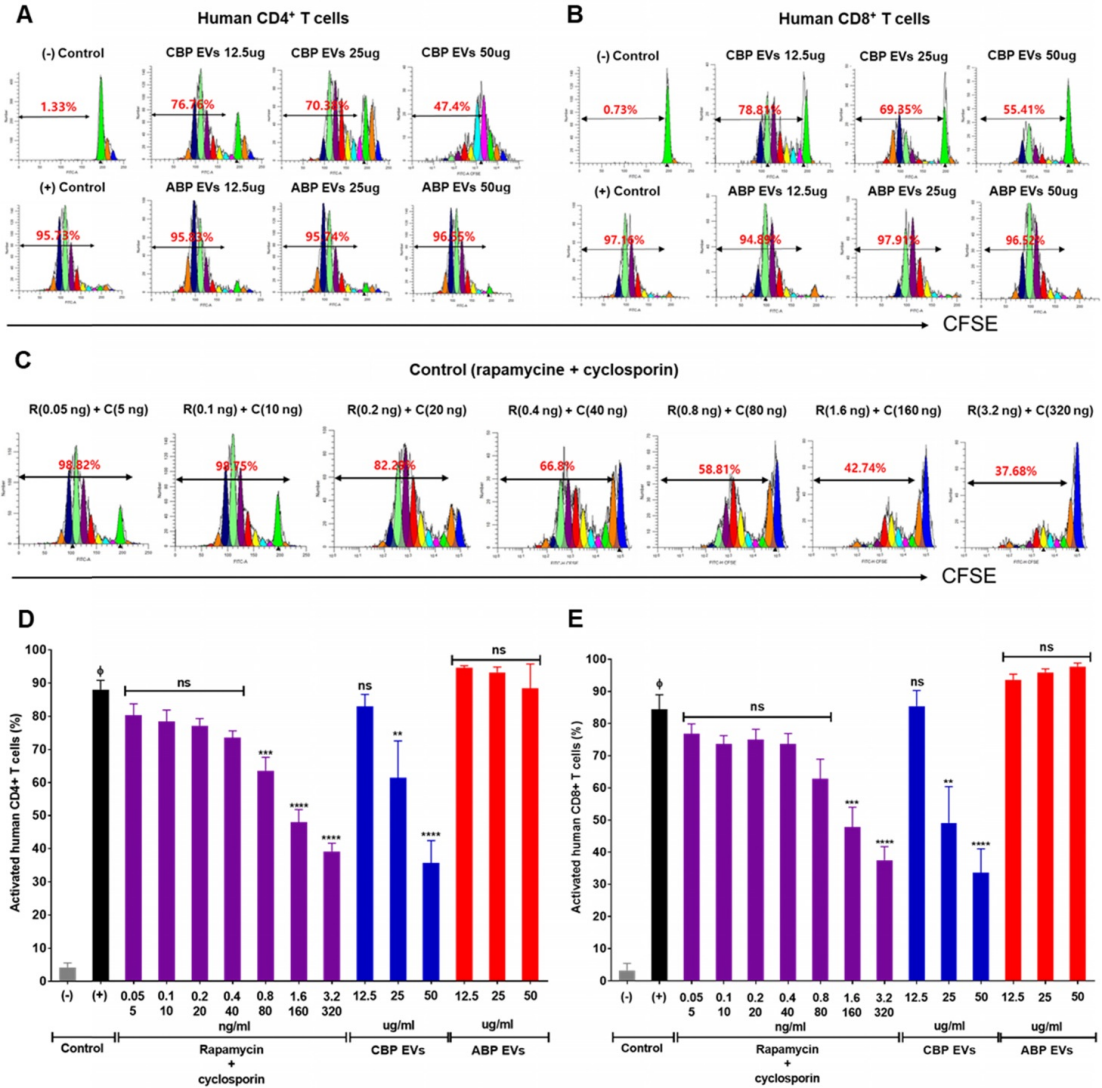
** Confirmation of CBP EV-mediated immunosuppression in stimulated human CD4^+^ and CD8^+^ T cells. (A-B)** Representative experimental data demonstrating that CBP EVs inhibited human CD4^+^ and CD8^+^ T cell stimulation. **(C)** The immunosuppressants, rapamycin and cyclosporine, were used as the standard positive controls. The intensity of CFSE-labeled T cells was acquired by flow cytometry and further analyzed using ModFit LT 4.0 software. **(D-E)** Human CD4^+^ and CD8^+^ T cells were activated using CD3/CD28 DYNABEAD in the presence of CBP EVs or ABP EVs dose-dependently. The intensity of CFSE-labeled T cells was acquired by flow cytometry and was further analyzed using ModFit LT 4.0 software. All experiments were repeated a minimum of five times using different batches of CBP EVs (one batch of plasma EVs = the sum of 10 units of cord blood). Statistically significant differences (ANOVA test): *p < 0.1, **p < 0.05, ***p < 0.01. Activated PBMCs indicate the positive control = Φ.

Rapamycin and cyclosporine are used as positive control for the inhibition of the immune response in a dose-dependent manner (Figure 2C). In the present study, we mainly focused on the mechanisms of immunosuppression mediated by direct contact between CBP EVs and immune cells. To address this, DYNABEAD anti-CD3/CD28-activated PBMCs were cultured with CBP EVs or ABP EVs to determine whether CBP EVs exhibit potent immunosuppressive effects on activated immune cells (Figure 2A-B). The immunosuppressive effects of CBP EVs were examined by CD4^+^ and CD8^+^ T cell proliferation. We found that CBP EVs, but not ABP EVs, suppressed CD4^+^ and CD8^+^ T cell proliferation, as repeatedly analyzed by the CFSE dilution assay (Figure 2D-E).

### Mechanisms of inhibiting human T cell proliferation of CBP EVs

**Figure 3:**
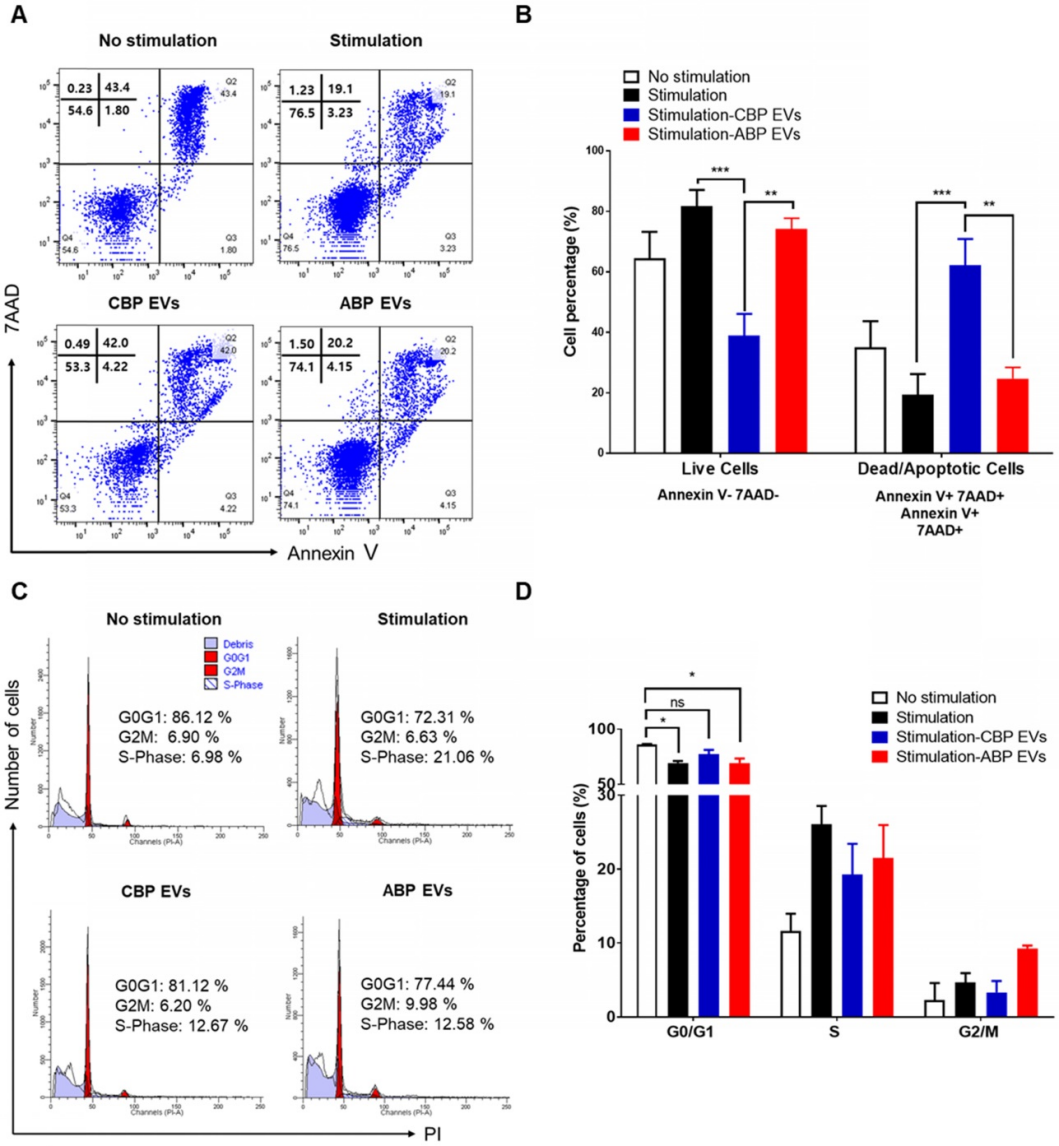
** Mechanism underlying CBP EV-mediated immunosuppression in CD3/CD28 Dynabead-stimulated human T cells. (A)** CBP EVs induced apoptosis of Human T cells activated by CD3/CD28 DYNABEADs. CD4^+^ T cells were harvested after 156 h and stained with Annexin V-FITC, 7AAD, anti-CD4-APC antibodies. The cells gated on CD3^+^ are shown. The assay data were analyzed using FlowJo v10. **(B)** This experiment was repeated five times independently. Statistically significant differences (ANOVA test): *p < 0.1, **p < 0.05, ***p < 0.01. **(C)** CBP EVs-induced G0/G1 cell cycle arrest of human CD3+ T cells was activated by CD3/CD28 DYNABEAD. Human CD3+ T cells were stimulated with DYNABEAD and CBP EVs were added at the same time. After 156 h of incubation, the cells were stained with propidium iodide for 20 min and assessed by flow cytometry to reveal the effect of CBP EVs on cell cycle progress. These data were analyzed using ModFit LT 4.0 software. **(D)** This experiment was repeated three times independently. One-way ANOVA was used to calculate the significance between groups: *p < 0.1 **p < 0.05 ***p < 0.01.

To delineate the underlying mechanisms of the immunosuppressive effect, we investigated the proportion of apoptotic cells in CBP EV- and ABP EV-treated groups with Annexin V and 7AAD via flow cytometry analysis. We also verified whether CBP EVs may influence the apoptosis of activated T cells. The results revealed that PBMCs were activated by DYNABEAD in the presence of EVs. Annexin V 7AAD double-negative staining indicated live cells, while 7AAD- and Annexin V-positive staining indicated dead and apoptotic cells, respectively. The results showed that in the CBP EV-treated group, the rate of apoptosis increased compared with that in the ABP EV-treated group. However, compared with the positive control, in the early apoptosis stage, the ABP EV-treated group did not show any differences (Figure 3A-B). We hypothesized that the reduction in proliferation may be due to the induction of cell cycle arrest. Therefore, cell cycle analysis was performed by measuring the DNA content in each cell group by flow cytometry after propidium iodide (PI) staining. PBMCs were stimulated with DYNABEAD in the presence of CBP EVs or ABP EVs and incubated for 156 h. Subsequently, the cells were stained with PI. Although the proportion of cells in the G0/G1 stage exhibiting apoptosis was higher in the CBP EV-treated group than in the control group, this difference was not significant. Furthermore, the frequency of cells in the G2/M and S phase was higher in the CBP ABP EV-treated group than in the control group at 156 h (Figure 3C-D). These results suggest that CBP EVs reduce T cell proliferation by inducing G0/G1 cell cycle arrest together with apoptosis.

### Comparative proteomic profile of human CBP EVs and ABP EVs

**Figure 4:**
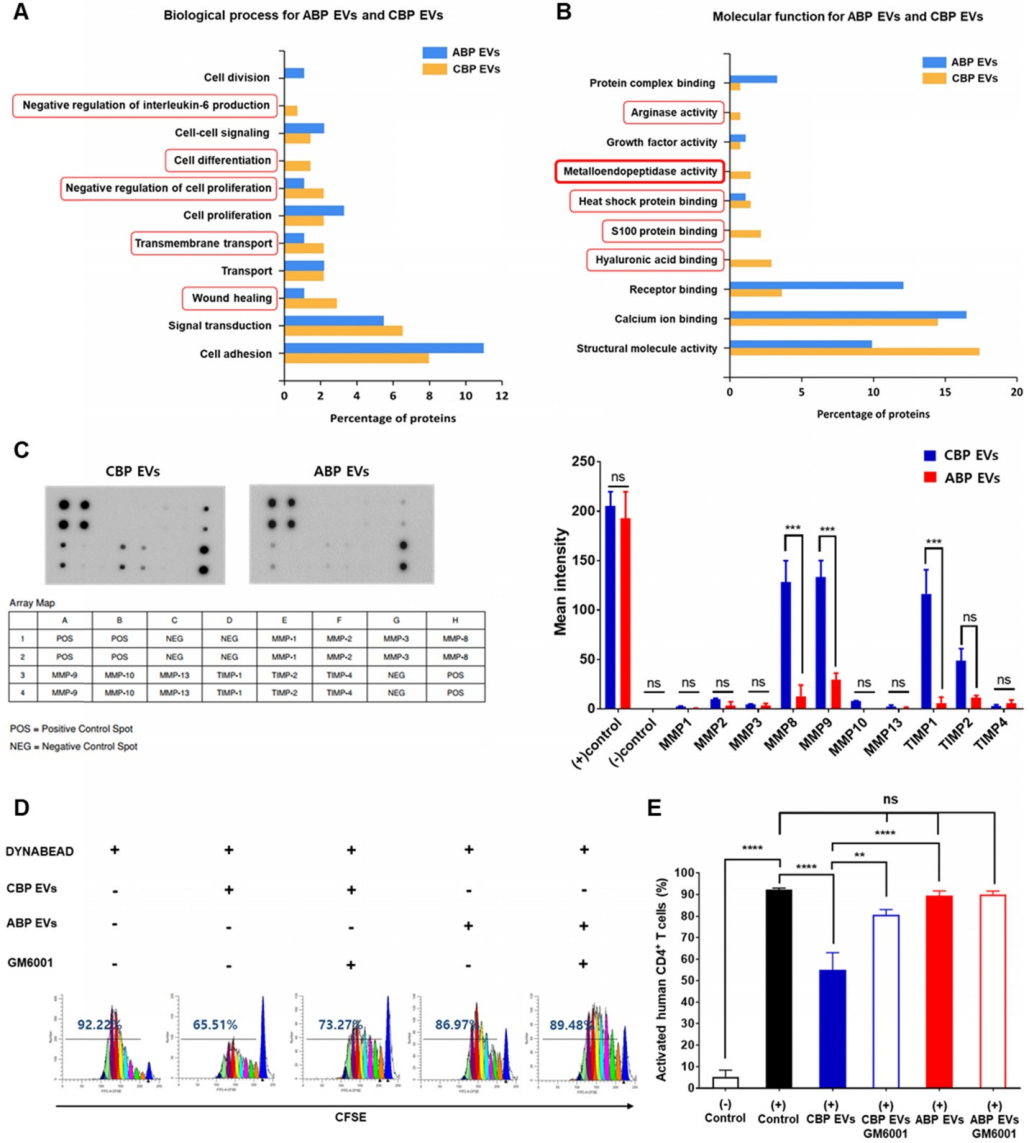
** Analysis of the differential molecular functions and biological processes of ABP EV and CBP EV, confirming the immunosuppressive response by MMP-expressing CBP EV. (A)** Proteomic analysis of various biological functions of human CBP EVs and ABP EVs using the ExoCarta database and SBC analysis system. **(B)** Proteomic analysis of human CBP EVs and ABP EVs, as well as various molecular functions of immunosuppression screened using the ExoCarta database and SBC Analysis system. The red box indicates a higher relevance of CBP EVs due to functional classification. **(C)** MMP and TIMP antibody arrays using 300 μg of CBP EV or ABP EV lysate; the corresponding dots were evaluated using an EV antibody array kit. The MMP and TIMP antibody spots that produced signals of varying intensities were calculated using ImageJ software. This experiment was repeated three times independently.** (D)** CBP EV-mediated suppression via MMP inhibition results in the partial restoration of T cell proliferation. T cells were stimulated with DYNABEAD in the presence of CBP EVs and then treated with or without GM6001 before comparison via CFSE analysis at 156 h. The CFSE-labeled cells were acquired by FACSCanto, and the cells were gated on CD4^+^ events. **(E)** The percentage of cells in each generation was calculated using ModFit LT 4.0 software. This experiment was repeated six times independently. Statistically significant differences (ANOVA test): *p < 0.1, **p < 0.05, ***p < 0.01.

The functional classification of ABP EVs and CBP EVs and further comparison between these EVs highlighted five distinct biological processes and molecular functions. Some of them are found exclusively within a particular pathway, such as negative regulation of IL-6, cell differentiation, negative regulation of cell proliferation, transmembrane transport, and wound healing (Figure 4A). Focusing on the molecular functions of ABP EVs and CBP EVs, we found interesting features associated with metalloendopeptidase activity. CBP EVs appear to be associated with arginase activity, heat shock protein binding, S100 protein binding, and hyaluronic acid binding to a higher degree than that of ABP EVs (Figure 4B). Specifically, proteomic analysis of CBP EVs revealed that exosomal proteins are associated with immunosuppression. Based on these findings, we selected proteins which are known to be related to immune system regulation to determine which factors were involved in impairing T cell proliferation. The functional classification of CBP EVs was performed using the ExoCarta database and GO analysis (Table 1).

### Proliferation of T cells restored by inhibition of MMP expressed in CBP EVs

We next sought to identify the factors in CBP EVs responsible for the inhibition of T cell proliferation. It has been reported that CD25 is proteolytically cleaved by MMP-9 and shows immunosuppressive effects on T cells 29, 37. These zinc-dependent endopeptidases are essential factors for cell invasion through the extracellular matrix. To determine the presence of MMPs in CBP EVs, we used a human MMP antibody array to analyze diverse MMPs and TIMPs in CBP EVs. As shown in Figure 4C, despite the expression of the MMP inhibition molecules TIMP1 and TIMP2, CBP EVs contained significantly elevated concentrations of MMP-9 and -8. Thus, we speculate that CBP EVs cleave membrane-bound CD25 via MMP-9 and inhibit the proliferation of activated T lymphocytes. For subsequent confirmation that MMP is involved in immunosuppression, CBP EVs were incubated with GM6001, an inhibitor of various MMPs, including MMP-1, -2, -3, -8, and -9, for 2 h before incubation with human T cells. Dose-dependent experiments were performed with MMP inhibitors (GM6001) at varying concentrations from 2.5 ug/mL - 20 ug/mL. Since no significant effect was observed above 10 µg /mL, the titration of GM6001 was fixed to 10 µg /mL (Figure S1). Human CD4^+^ T cells cultured with GM6001-treated CBP EVs showed restored proliferation (Figure 4D-E). These results indicate that MMP-9 is mainly involved in immunosuppression of CBP EVs, which is caused by the inhibition of CD25 by MMP-9. Despite these results, the inhibition of MMP-9 does not seem to fully restore immune activity, suggesting the existence of additional mechanisms or the involvement of different proteins.

### Mouse *in vitro* analysis confirmed immunosuppressive function of CBP EVs

**Figure 5:**
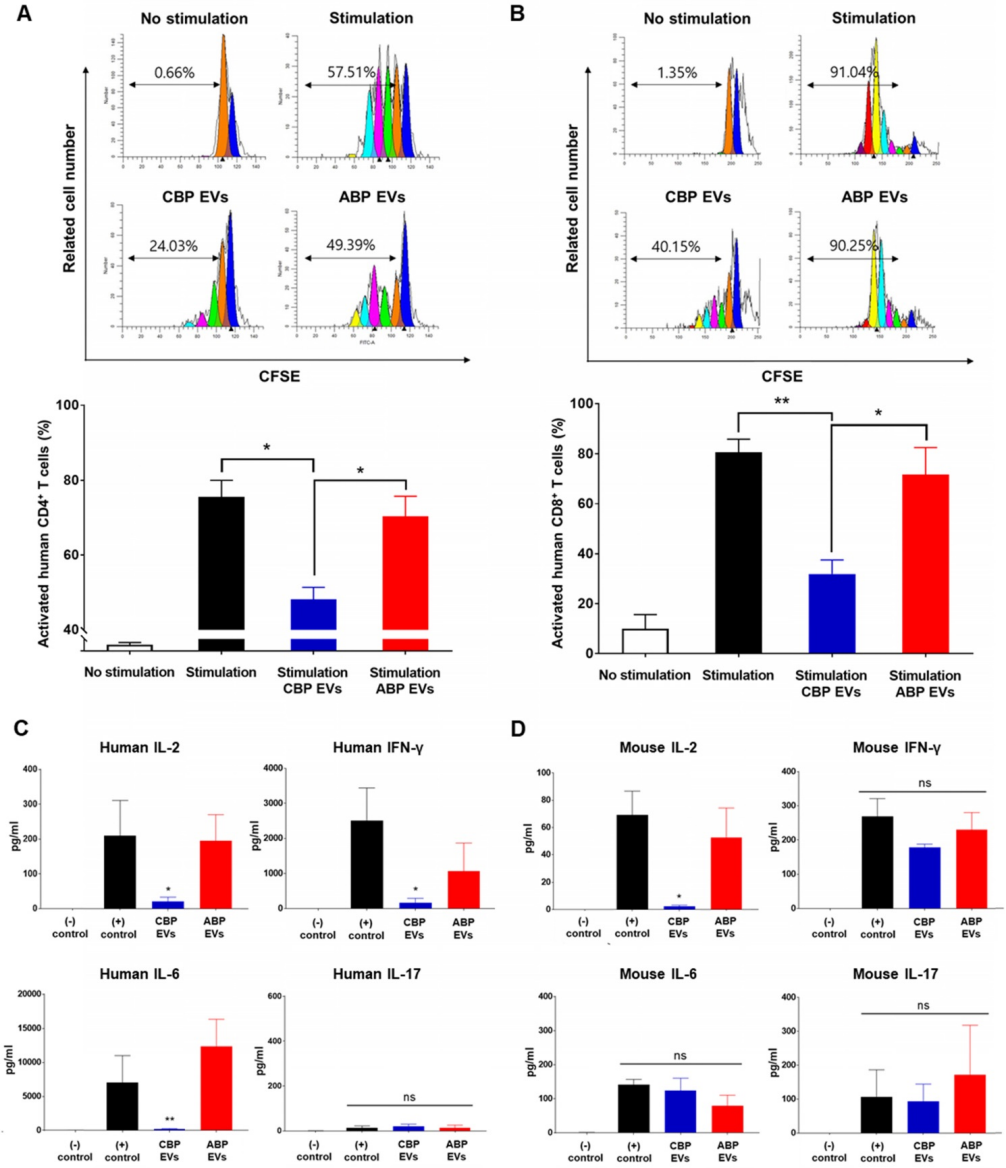
** Mouse cross-reactivity of T cell suppression and IL-2 downregulation by human CBP EVs. (A)** Immunosuppressive effects of CBP EVs were examined by DYNABEAD-stimulated mouse CD4^+^ T cell proliferation. This experiment was repeated five times independently. Statistically significant differences (ANOVA test): *p < 0.1, **p < 0.05, ***p < 0.01. **(B)** Immunosuppressive effects of CBP EVs were examined by DYNABEAD-stimulated mouse CD8^+^ T cell proliferation. This experiment was repeated five times independently. Statistically significant differences (ANOVA test): *p < 0.1, **p < 0.05, ***p < 0.01. **(C)** CBP EVs significantly decreased human T cell proliferation and downregulated IL-2, IFN-γ, and IL-6 secretion, which are associated with the differentiation of Th17 and Th1 cells. The cytokine level at 156 h in the harvested culture supernatant was analyzed using the human cytometric bead array. This experiment was repeated three times independently. **(D)** CBP EVs not only downregulated IL-2 secretion in human T cells but also reduced IL-2 in mice. The changes in cytokine levels were observed using the mouse cytometric bead array. This experiment was repeated three times independently.

Since EVs are derived from human UCB, it is important to demonstrate that mouse spleen cells have immunosuppressive functions in addition to human PBMCs *in vitro*. Therefore, after confirming the immunosuppressive ability of CBP EVs against T-cells in mouse splenocytes *in vitro*, the immunosuppressive ability of EVs was tested in preclinical animal models with autoimmune diseases. Human MMP9, which plays a major role in the immunosuppressive function of CBP EVs, can act on both humans and mice because mouse MMP-9 shares 99% homology with human MMP-9 38. In support of this finding, we discovered that CBP EVs exhibited a potent immunosuppressive effect on mouse splenocytes and human PBMCs. Immunosuppressive effects of CBP EVs were examined using DYNABEAD-stimulated mouse CD4^+^ and CD8^+^ T cell proliferation. Supporting our previous results, CBP EVs, but not ABP EVs, suppressed CD4^+^ and CD8^+^ T cell proliferation, as analyzed by the CFSE dilution assay (Figure 5A-B). *In vitro* analysis of this natural inhibition of human and mouse immune cells suggest that it may also occur in mouse disease models.

### CBP EVs modulate IL-2 signaling and Th1 and Th17 cell-related cytokines

We then cultured the cells with anti-CD3/CD28 for 6 days, allowing the T cells to expand and respond to CBP EVs. The cells from each well were washed and rested before their use in the ELISPOT assay. The results showed that the proportions of IL-2- and IFN-γ-secreting cells were markedly decreased when co-cultured with CBP EVs, while IL-17-secreting cells did not differ among the control, CBP EV-treated, and ABP EV-treated groups (Figure S5A-C). Thus, CBP EVs exhibited immunosuppressive functions by inhibiting IL-2- and IFN-γ-secreting cells. To assess cytokine secretion, the levels of IL-2, IFN-γ, and IL-6 were measured using the cytometric bead array. The results showed that CBP EVs downregulated human IL-2, IFN-γ, and IL-6, which are associated with the differentiation of Th1 and Th17 cells and the development of autoimmune diseases (Figure 5C). However, evaluation of the culture supernatant showed that the concentration of IL-2 alone was significantly reduced when cultured with CBP EVs in mouse T cells (Figure 5D). These data indicate that the inhibition of IL-2 production by CBP EVs is a crucial mechanism of T cell suppression in both human and mice.

### CBP EVs regulate IL-2 cytokines to alleviate EAE symptoms

**Figure 6:**
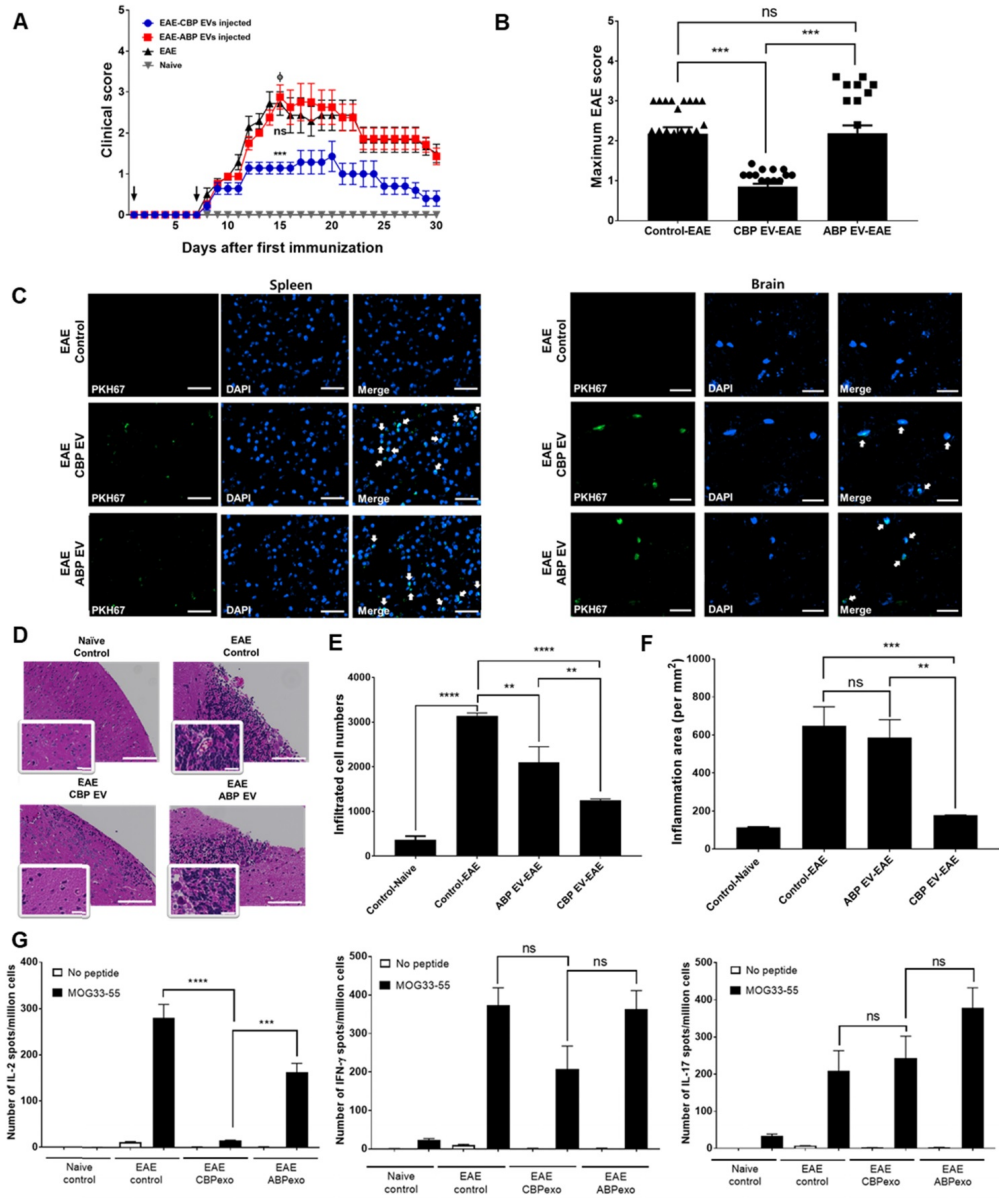
** Changing patterns of IL-2 levels in experimental autoimmune encephalomyelitis (EAE) mice treated with EVs. (A)** Development of EAE was reduced in CBP EV-treated EAE mice. EAE was induced in 15 C57BL/6 mice following co-treatment with MOG/CFA and PTx immunization. CBP EVs and ABP EVs were injected intravenously twice on days 0 and 7 into five EAE mice at a 100-μg dose (black arrow). The mice were assigned to different groups to analyze the clinical score after EAE induction: EAE mice (n = 5) versus CBP EV- or ABP EV- injected EAE mice (n = 5). EAE indicates positive control = Φ. **(B)** The maximum and minimum scores for each EAE group are shown. **(C)** Representative micrographs of the brain and spleen 24 h after injection of PKH67-stained EVs into the tail vein. EAE mice without EVs were used as the negative control. Blue - DAPI and green - PKH67-stained EVs. All scale bars are 20µm. **(D)** Hematoxylin and eosin (H&E) staining on the brain section of EAE induced animals which treated by EVs. Digital images were collected under a color bright-field setting using a ×20 objective. Scale bars represent 100 µm. **(E-F)** Quantitative comparison of the levels of cellular infiltration number and inflammation area within the brain sections from EAE mice treated with EVs. Images were processed using Gen5 software (n = 3). **(G)** Detection of MOG35-55-induced cytokine recall response in whole splenocytes during acute EAE. C57BL/6 mice were immunized with MOG33-55 in CFA and tested 22 days after immunization. All tested mice exhibited clinical symptoms of EAE. The frequency of MOG peptide-specific IFN-γ-, IL-2-, and IL-17-secreting cells was determined for comparison with that of the EAE control, CBP EV-treated EAE, and ABP EV-treated EAE groups using the ELISPOT assay. MOG peptide-specific T cells were analyzed during re-stimulation with MOG peptides (25 μg/mL) (n = 3).

To confirm this result in alternate models of EAE treated with CBP EV, we first examined whether prophylactic (pre-onset) treatment with CBP EV could prevent EAE induction. The clinical scores of the CBP EV-treated EAE group were significantly lower than those of the ABP EV-treated EAE group and the EAE control. These results reveal that prophylactic treatment with CBP EV could significantly alleviate the induction of progressive EAE (Figure 6A-B). Next, to evaluate the therapeutic efficacy of CBP EV in progressive EAE, we examined whether post-onset CBP EV treatment for advanced EAE could mitigate the severity of EAE. However, in an advanced stage of EAE, we were unable to observe a significant therapeutic effect of CBP EV treatment (Figure S3A). The maximum disease scores for each EAE group are shown in Figure S3B. These data indicate that CBP EV treatment can suppress progressive EAE more efficiently when initiated at an early stage of the disease. PKH67-labeled EVs (green fluorescence) were injected into the tail vein of EAE mice. Sections of brain and spleen tissue were counter-stained with DAPI for localization of cell nuclei and observed under a Lionheart imaging system (BioTek Instruments, Winooski, VT). PKH67-labeled EVs were detected in both the spleen and brain tissue (Figure 6C). Histopathology examination revealed that CBP EV prophylactic treatment reduced the number of cellular infiltrations compared with EAE and ABP EV-treated EAE mice (Figure 6D-E). The inflammatory cell area was also significantly reduced in the brain of CBP EV treatment groups compared to the EAE and ABP EV treated group (Figure 6F). We next verified whether infused CBP EVs have an immunoregulatory function in helper T cells. To this end, we measured the expression level of cytokines in splenocytes using the ELISPOT assay. Moreover, the frequency of MOG peptide-specific IFN-γ-, IL-2-, and IL-17-secreting cells was determined for comparison among the EAE control, CBP EV-treated EAE, and ABP EV-treated EAE groups by the ELISPOT assay. MOG peptide-specific IL-2 cells were significantly reduced in the CBP EV-treated EAE group. However, similar to the results of the *in vitro* analysis, no significant difference was observed in IFN-γ- and IL-17-secreting cells (Figure 6G). Donor CD4^+^ T cells reciprocally differentiate into Th1 and Th17 cells that mediate EAE and the balance of the Th subset, playing a pivotal role in regulating the T cell immune response. We further investigated their ability to reduce Th1 and Th17 cell-related cytokines by CBP EVs. Densitometric evaluation of the secreted proteins indicates that administration of CBP EVs triggered a decrease in the levels of IL-2, and IFN-γ compared with those in the EAE group (Figure S5D-F). However, for alleviating EAE symptoms, IL-2 cytokine regulation by CBP EVs seems to have the most important effect. CBP EV injection significantly suppressed disease progression in the early stages of EAE, but IL-2 reduced the inhibitory effect after the onset of EAE disease, because IL-2 affects the early stages of T cell development.

### CBP EV induce immune regulatory cell differentiation in the EAE model

**Figure 7:**
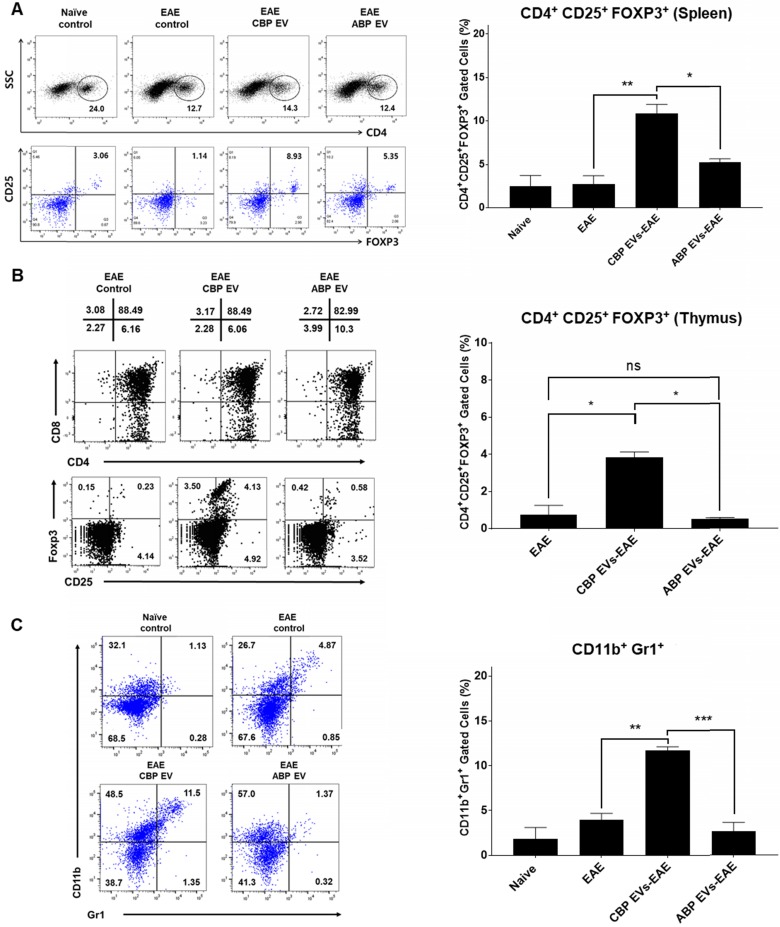
** Changing patterns of Treg and MDSC levels in experimental autoimmune encephalomyelitis (EAE) mice treated with EVs. (A)** The Treg population in EVs-treated EAE mice splenocytes. The frequency of CD4^+^ gated MOG peptide-specific FOXP3^+^ cells was determined and compared with that of the EAE control, CBP EV-treated EAE, and ABP EV-treated EAE groups by intracellular cytokine staining (n = 3). **(B)** Expression profiles of CD4 versus CD8 and Foxp3 versus CD25 (gated on CD4^+^ T cells) for thymocyte populations in EAE, CBP EV injected EAE, ABP EV injected EAE (n=3). **(C)** MDSC populations in EAE mice treated with EVs. The frequencies of CD11b^+^Gr1^+^ cells were determined or compared with those of the MDSC population in the healthy control, EAE control, CBP EV-injected EAE, and ABP EV-injected EAE groups (n = 3).

The frequency of CD4^+^/CD25^+^/FOXP3^+^ Tregs in splenocytes was determined and compared among the non-treated control (negative control), EAE control (positive control), CBP EV-treated EAE, and ABP EV-treated EAE groups. We observed that the Treg population increased in the CBP EV-treated EAE group compared with that in the control groups (Figure 7A). These results demonstrate that CBP EVs exert immunosuppressive effects by inhibiting the IL-2 signaling pathway and by increasing the proportion of CD4^+^/CD25^+^/FOXP3^+^ Treg cells *in vivo*. The total thymic cellular frequencies of CD4- CD8-, CD4+ CD8+, CD4+ CD8+, and CD4- CD8+ thymic cells are similar for EAE, CBP EV-injected EAE, and ABP EV-injected EAE mice. However, in the EAE or ABP EV-injected EAE mice, the ratio of CD4+ CD25+ Foxp3+ thymic cells were 0.23-1.25%, whereas in the CBP EV-injected EAE mice the ratio was comparatively higher at 3.53-4.13% (Figure 7B). Furthermore, the CD11b^+^/Gr-1^+^ cell phenotypes (mouse MDSCs) were expanded during EAE disease progression in CBP EV-injected mice (Figure 7C). This suggest that MDSCs became enriched and accumulated in splenocytes of CBP EV-injected EAE mice may play a regulatory role.

### Transfer of GAL-3, GAL-7, S100-A7, MMP-9, MMP-8, HSP-72, and PIP genes into the H1ME-5 cell line to generate CBP EV mimics

**Figure 8:**
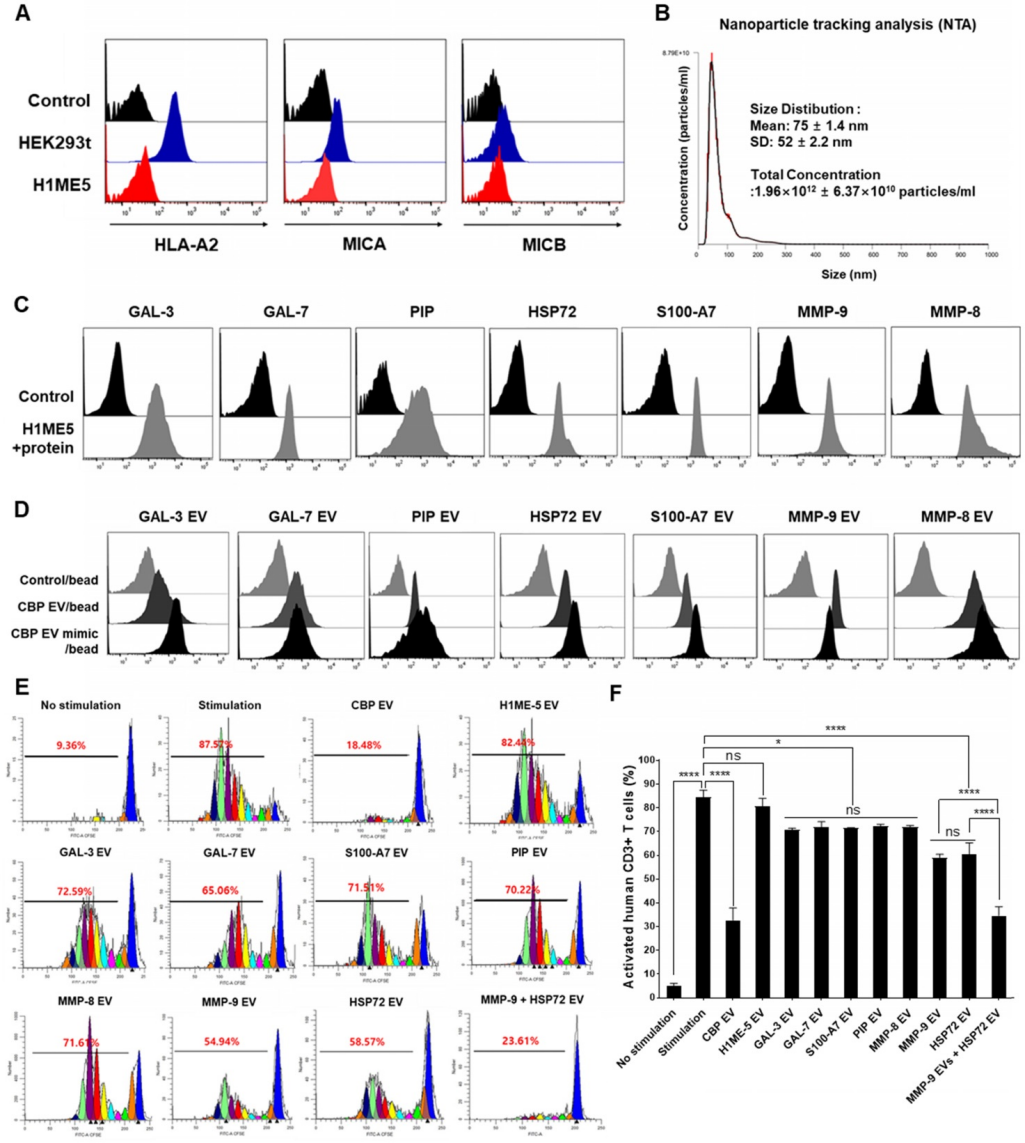
** CBP EV mimics enriched in MMP-9 and HSP-72 molecule exhibit immunosuppressive effects. (A)** The establishment of the human leukocyte antigen (HLA) class I/MIC null HEK 293T (H1ME-5) cell line using the multiplex CRISPR/Cas9 system. H1ME-5 cells show no expression of MICA/B and HLA class I. **(B)** The particle number of H1ME-5 EVs was 1.96×10^12^ ± 6.37×10^10^ particles/mL. Representative NTA data indicating the size of H1ME-5 EVs (75 ± 1.4 nm). **(C)** Expression of the CBP EV-contained molecules GAL-3, GAL-7, S100-A7, MMP-9, MMP-8, HSP-72, and PIP transduced into H1ME-5 cells. Six days following transduction, cells positive for each molecule were sorted using a MoFlo XDP Cell Sorter. **(D)** CBP EVs or CBP EV mimics bound to latex beads coated with anti- GAL-3, GAL-7, S100-A7, MMP-9, MMP-8, HSP-72, and PIP antibodies were analyzed by flow cytometry. Intensities derived from GAL-3, GAL-7, S100-A7, MMP-9, MMP-8, HSP-72, and PIP with corresponding bead-only controls. **(E)** Representative experimental data demonstrating that CBP EV, MMP-9-, and HSP72- EV inhibited T cell stimulation. The intensity of CFSE-labeled CD3^+^ T cells was acquired by flow cytometry and further analyzed using the ModFit LT 4.0 software. **(F)** H1ME-5 EVs showed no immunosuppressive effect; only HSP72, MMP-9 expressing EV showed significant immunosuppression. The combination of EV expressing MMP-9 and HSP72 reached an immunosuppressive effect similar to that of CBP EV. The combination of EVs expressing MMP-9 and HSP72 showed a more significant effect than when using HSP72 and PIP alone. The statistical values ​​were analyzed using the results obtained from the CFSE proliferation assay. (ANOVA test): *p < 0.1, **p < 0.05, ***p < 0.01, ****p < 0.001.

Focusing on the molecular functions of CBP EVs, we identified interesting features associated with GAL-3, GAL-7, S100-A7, MMP-9, MMP-8, HSP-72, and PIP, which are associated with immune regulation (Table 1). MICA induces natural killer cell proliferation, and MHC-I deficiency compromises immune activation. Therefore, H1ME-5 cells were silenced using the CRISPR/cas9 system to generate cells lacking immune-activation effects. H1ME-5 cells are suitable for producing CBP EV mimics since H1ME-5 cell-derived EVs exhibit no immunosuppressive effect (Figure 8E-F). For complete elimination of the HLA class I and MICA/B genes, we used seven plasmids that encode the Cas9 protein and gRNAs to target exons 2 and 3 of the HLA-A, HLA-B, HLA-C, and MICA/B genes as described previously 35. The H1ME-5 cells exhibiting a lack of HLA class I and MICA/B expression were analyzed using flow cytometry (Figure 8A) and transduced with lentiviruses encoding GAL-3, GAL-7, S100-A7, MMP-9, MMP-8, HSP-72, and PIP genes. Six days following transduction, GAL-3, GAL-7, S100-A7, MMP-9, MMP-8, HSP-72, and PIP positive cells were sorted. H1ME-5 cells expressed GAL-3, GAL-7, S100-A7, MMP-9, MMP-8, HSP-72, and PIP at a rate higher than 90% by flow cytometry (Figure 8C). In addition, EVs derived from this cell line were analyzed by flow cytometry to confirm that the target molecules GAL-3, GAL-7, S100-A7, MMP-9, MMP-8, HSP-72, and PIP were sufficiently expressed (Figure 8D). Malvern Zetasizer Nano ZS (DLS) was used to measure approximate sizes, and the Nanosight NS 300 system (NTA) was used to more accurately determine sizes and to determine the EV concentration from the number of nanoparticles. The particle number and size of H1ME-5 EVs, measured using NTA, were 1.96×10^12^ ± 6.37×10^10^ particles/mL and 75 ± 1.4 nm (Figure 8B). The particle size of artificial EVs were measured using DLS 145.61 ± 75.89 nm and 1.64 × 10^12^ ± 2.6 × 10^10^ particles / mL, respectively, with no difference between each of the seven EVs (Figure S4). Of the seven candidate EVs, those expressing MMP-9 and HSP-72 showed immunosuppressive effects, and the combination of EVs expressing MMP-9 and HSP72 exerted immunosuppressive effects similar to those of CBP EV (Figure 8E-F). Furthermore, the EV combination expressing MMP-9 and HSP72 exhibited higher immunosuppressive effects compared with that of HSP72 and PIP alone (Figure 8E-F). The differential expression level of proteins in CBP EVs is highlighted in color in Table 1; in red, is an EV protein that affects immunosuppression at a high level, and in blue, it is a protein showing low immunosuppressive effects.

These data indicate that MMP-9 plays a vital role among the various immunosuppressive molecules present in CBP EVs. The immunosuppressive effect of each engineered H1ME-5 EV on CD4^+^ and CD8^+^ T cells was not significantly affected. However, the combination of engineered H1ME-5 EVs expressing MMP-9 and HSP-72 was found to increase activated T cell inhibition. Additionally, combinations of EVs expressing additional immunosuppressive proteins are expected to exhibit immunosuppressive capacities similar to CBP EVs.

## Discussion

Several proteins in CBP EVs have been reported to be associated with immunosuppression 24. In the present study, we found that CBP EVs contain more immunosuppression-related proteins than ABP EVs (Figure 4A-B and Table 1). The suppression of T cell proliferation by factors in CBP EVs was due to apoptosis and cell cycle arrest (Figure 3). It has been postulated that CBP EVs cleave CD25, which inhibits downstream mTOR signaling 39, resulting in cell cycle arrest. Indeed, the secretion of IL-2 and surface expression of CD25 in CBP EV-treated activated T cells were significantly reduced in CD4^+^ T cells (Figure 5C-D, Figure S5A, Figure S2). Furthermore, blockade of IL-2 signaling by CBP EVs plays a central role in the inhibition and proliferation of CD4^+^ and CD8^+^ T cells. Inhibiting the IL-2 signaling pathway inhibits the immune system, thus exerting therapeutic effects. The IL-2/IL-2R interaction activates the intracellular Ras/Raf/MAPK, JAK/STAT, and PI3K/AKT signaling pathways, stimulating the growth, differentiation, and survival of T cells 40, 41.

In the present study, we investigated the inhibition of IL-2 signaling by CBP EVs in association with MMPs. Analysis of CBP EV lysates with the human MMP antibody array confirmed the presence of significant concentrations of MMP-8 and -9 (Figure 4C). Elastases and proteases such as MMP-9 are known to induce the enzymatic cleavage of membrane-associated CD25 42, 43. Furthermore, MMP-9 is involved in the cleavage of CD25, which is expressed on T cells upon encountering cancer cells 44. Moreover, MMP-8 also induces the breakdown of extracellular domains 45, leading to the cleavage of surface molecules associated with immune responses, thus demonstrating the immunomodulatory effect of MMP on immune cells 29. The functional role of MMP-9 in the cleavage of CD25 was also confirmed by blocking analysis using GM6001, an inhibitor of various MMPs, including MMP-1, -2, -3, -8, and -9 (Figure 4D). MMP-9 may also play an important role in the preventive effects of EAE, and it has been detected in CSF, serum, and lesions in MS patients 46. MMP-9 is associated with leukocyte migration and destruction of the blood-brain barrier (BBB) 46. EVs can also spread through the blood-brain barrier (BBB), which is known to occur when there is an inflammatory condition such as EAE 47, 48. Thus, CBP EVs expressing MMP-9 may pass through the BBB of the EAE and have a direct therapeutic effect.

Upon employing human CBP EVs in mouse models, the mouse cross-reactivity of human CBP EVs immunosuppressive effects was also confirmed in mouse T cells *in vitro* (Figure 5A-B). The EAE mouse model has been extensively used to investigate the role of Th cells in disease development. For instance, transferring myelin-specific CD4^+^ Th1 cells to immature recipient mice reportedly induces EAE 49-55. Similarly, IL-17-secreting T cells (Th17 cells) are the driving force behind EAE development 56. Further, EAE promotes the onset of diseases by lowering the frequency of Tregs or impairing their inhibitory function 57. Thus, the *in vivo* expansion of Tregs has the potential to treat autoimmune diseases 58. Our results demonstrated that CBP EVs could alter the course of EAE development by the regulation of cytokine, Treg, and MDSC *in vivo* (Figure 6E, Figure 7). However, CBP EVs significantly inhibited IFN-γ, IL-2, and IL-6 expression in human T-cells but did not show an equally potent inhibitory effect in mouse T-cells due to xenogenic problems (Figure 5C-D, Figure 6E). Notably, IL-2 expression was significantly inhibited in both humans and mice. Since IL-2 expression affects the early stages of T-cell development, the results of animal experiments confirmed that CBP EV injections significantly inhibited EAE at the early stage but exhibited no suppressive effect after its onset.

Since only a limited supply of UCB exists, and it is difficult to isolate sufficient EVs for in-depth studies, these proteins were expressed in EVs to create CBP EV mimics. Based on the proteomics analysis, specific essential proteins, found to be abundant in CBP EVs and involved in immunosuppression, were selected. GAL-3 modulates immune escape of tumor cells by targeting the survival of effector CTLs and Th1 cells, or by skewing the balance toward a Th2-type cytokine profile and/or inducing the differentiation and expansion of Tregs 59-61. Hsp-72 plays a role in immunosuppression through the recruitment and activation of Tregs and MDSCs, leading to the downregulation of T cell responses 40. Hsp-72 also promotes immunosuppression via the activation of MMP-2 and production of MMP-9 62. Umbilical CBP contains a high concentration of MMP-9, which cleaves CD25 to inhibit T cell immunity 25, 29. PIP expression is associated with decreased cell proliferation and an increase of the apoptotic pathway 63. Many of the genes affected by PIP appear to be regulated by STAT5, which is associated with Treg differentiation 64, 65.

EVs isolated from H1ME-5 cells are suitable for genetic engineering as these cells lack immunosuppressive properties (Figure 8E-F). For this reason, CBP EV mimics were constructed using the H1ME-5 cell line, which express GAL-3, GAL-7, S100-A7, MMP-9, MMP-8, HSP-72, and PIP proteins, EVs were then isolated and characterized in this cell line (Figure 8C-D). Our result shows that MMP-9 and HSP-72-enriched EVs, one of the CBP EV mimics, represent promising therapeutic molecules for T cell immunosuppression (Figure 8E-F). Our results also indicated that several compounding effects, such as the cleavage of CD25 by MMPs, inhibition of IL-2 production, changes in cytokine secretion patterns, and proliferation of Treg cells, were simultaneously involved in T cell differentiation and proliferation. In addition, EVs expressing HSP-72 inhibited T cell proliferation, and HSP-72 is known to upregulate MMP-9, as described above. Consequently, it was confirmed that the combination of EVs expressing HSP-72 and MMP-9 most effectively enhances immunosuppressive effects. However, the composition of CBP EVs is extremely complex, and a broad spectrum of proteins is expected to function as immunosuppressants. Therefore, in future studies, we will examine the mechanism of immunosuppression by producing a recombinant variant of EVs with a more diverse protein combination.

## Conclusion

CBP EVs inhibit T cell proliferation while inducing Treg and MDSC differentiation. The immunosuppressive mechanism of CBP EV is likely due to the inhibition of IL-2 signaling by MMP-9. However, further inhibition of MMP-9 did not result in the complete recovery of proliferation, and thus, further studies on the underlying mechanisms of action of other molecules involved in immunosuppression are warranted.

## Supplementary Material

Supplementary figures.

## Figures and Tables

**Table 1:** The functional classification of immune regulation-associated CBP EV proteins.

Immune regulation associated proteins in CBP EV	Molecular Function	Biological process
**Heat shock 72 kDa protein 1A OS=Homo sapiens GN=HSPA1A PE=1 SV=1**	**Receptor binding**	**Cell-cell adhesion, Regulation of cell death**
**Matrix metalloproteinase-9 OS=Homo sapiens GN=MMP9 PE=1 SV=2**	**Metallopeptidase activity**	**Cytokine-mediated signaling pathway**
*Neutrophil collagenase OS=Homo sapiens GN=MMP8 PE=1 SV=2*	*Metallopeptidase activity*	*Positive regulation of nitric oxide biosynthesis*
*Galectin-3-binding protein OS=Homo sapiens GN=LGALS3 PE=1 SV=1*	*Immune regulator molecules*	*Receptor-mediated endocytosis*
*Prolactin-inducible protein OS=Homo sapiens GN=PIP PE=1 SV=1*	*IgG binding*	*Regulation of T cell apoptotic process*
*Galectin-7 OS=Homo sapiens GN=LGALS7 PE=1 SV=2*	*Immune regulator molecules*	*Receptor-mediated endocytosis*
*Protein S100-A7 OS=Homo sapiens GN=S100A7 PE=1 SV=4*	*Zinc ion binding*	*Angiogenesis, Apoptotic process*
Arginase-1 OS=Homo sapiens GN=ARG1 PE=1 SV=2	Arginase activity	Regulation of cell proliferation
Peroxiredoxin-1 OS=Homo sapiens GN=PRDX1 PE=1 SV=1	Cadherin binding involved in cell-cell adhesion	Cell proliferation, Natural killer cell mediated cytotoxicity
Proteasome subunit alpha type-6 OS=Homo sapiens GN=PSMA6 PE=1 SV=1	NF-kappaB binding	Regulation of inflammatory response
Lysosome-associated membrane glycoprotein 1 OS=Homo sapiens GN=LAMP1 PE=1 SV=3	Protein binding	Granzyme-mediated apoptotic signaling pathway
Serpin B12 OS=Homo sapiens GN=SERPINB12 PE=1 SV=1	Serine-type endopeptidase inhibitor activity	Regulation of cell proliferation
Lactotransferrin OS=Homo sapiens GN=LTF PE=1 SV=6	Heparin binding	Regulation of apoptotic process
Alpha-1-acid glycoprotein 1 OS=Homo sapiens GN=ORM1 PE=1 SV=1	Protein binding	Negative regulation of tumor necrosis factor production
CD5 antigen-like OS=Homo sapiens GN=CD5L PE=1 SV=1	Scavenger receptor activity	Apoptotic process
Mannan-binding lectin serine protease 1 OS=Homo sapiens GN=MASP1 PE=1 SV=3	Calcium ion binding	Proteolysis

The bolded and italicised proteins are a protein expressed in artificial EV, the EV protein bolded is an EV protein that affects immunosuppression at a high level, and the EV protein italicised is a protein showing low immunosuppressive effect.

## Abbreviations

UCB: Human umbilical cord blood
EVs: extracellular vesicles
CBP EVs: UCB plasma-derived extracellular vesicles
ABP EVs: adult blood plasma-derived extracellular vesicles
PBMC: Human peripheral blood mononuclear cells
EAE: experimental autoimmune encephalomyelitis
MMP-9: matrix metallopeptidase
Tregs: regulatory T cells
MDSCs: myeloid-derived suppressor cells
H1ME-5: HLA class I and MICA/B edited clone-5
PVDF: polyvinylidene fluoride
CFSE: carboxyfluorescein diacetate-succinimidyl ester
ELISPOT: enzyme-linked immunospot
MOG: myelin oligodendrocyte glycoprotein
LC-MS/MS: Liquid chromatography with tandem mass spectrometry
