# Supplementary material for: Extracellular vesicles from human umbilical cord blood plasma modulate interleukin-2 signaling of T cells to ameliorate experimental autoimmune encephalomyelitis
Source: Theranostics. 2020 Apr 6;10(11):5011–28. doi: 10.7150/thno.42742 (PMC7163430; doi:10.7150/thno.42742)
Supplement: Supplementary file 1 — Supplementary figures. [file thnov10p5011s1.pdf]

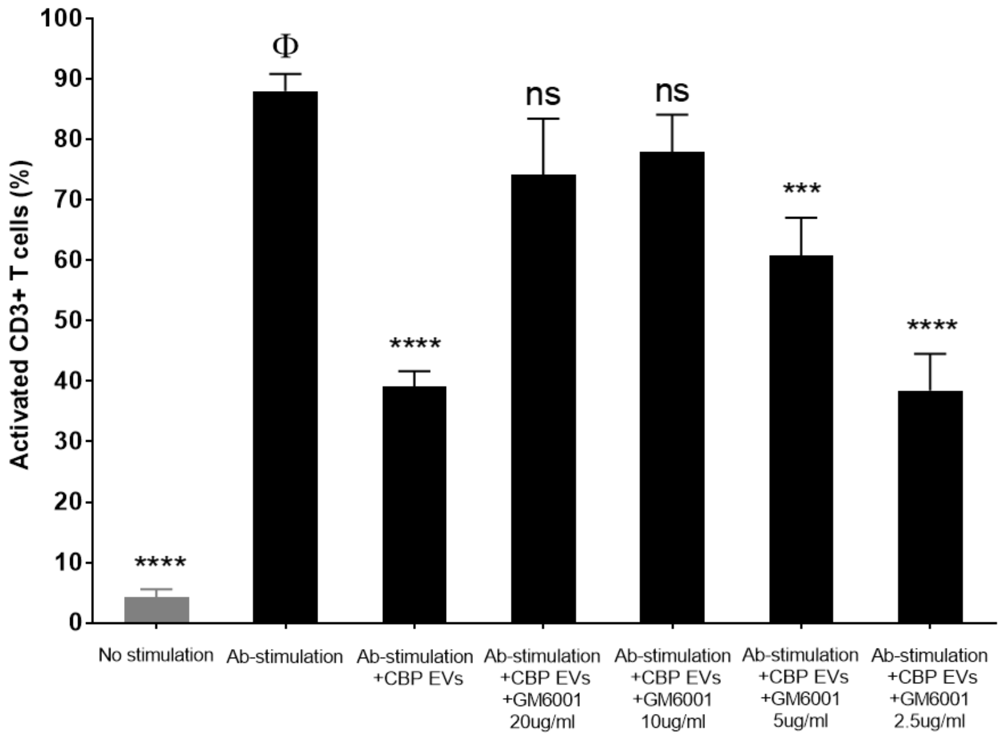

Figure S1. Human PBMCs were activated using CD3/CD28 DYNABEAD in the presence of CBP EV to dose-dependently inhibit MMPs, thereby confirming the recovery of immune activation of T cells. Activated PBMCs indicate the positive control = Φ.

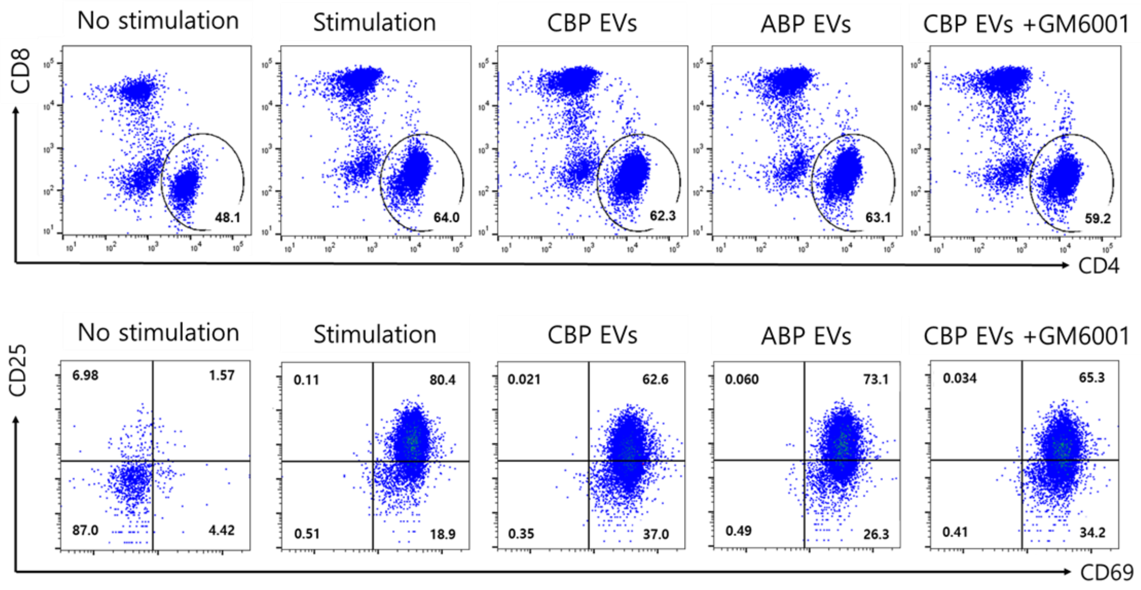

Figure S2. CBP EVs downregulated the expression of activation marker CD25 but not CD69.

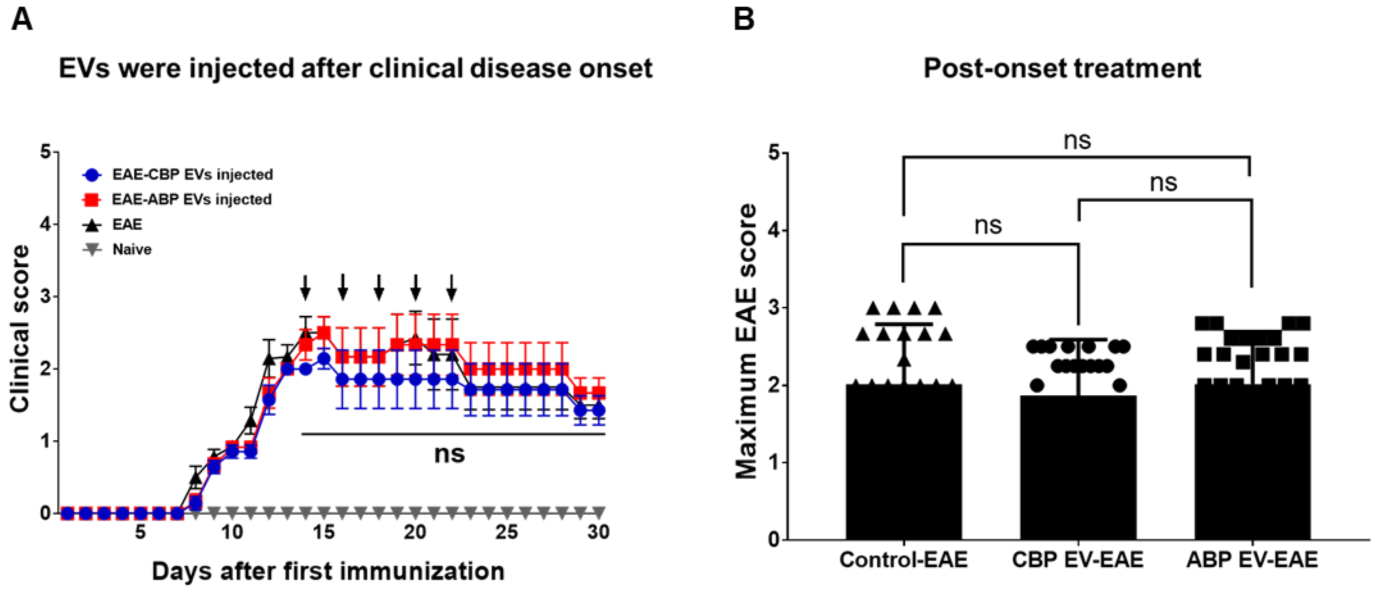

Figure S3. (A) After disease onset, the CBP EV injection did not have a significant effect on EAE. To properly treat the mice at various disease stages, the mice that exhibited early clinical signs of EAE (disease grade 2–3) were provided late CBP EV treatment every alternate day for 10 days (n = 5). (B) The maximum and minimum scores for each EAE group are shown. All disease scores for each EAE groups were represented as the daily scores for the 30-days experiment.

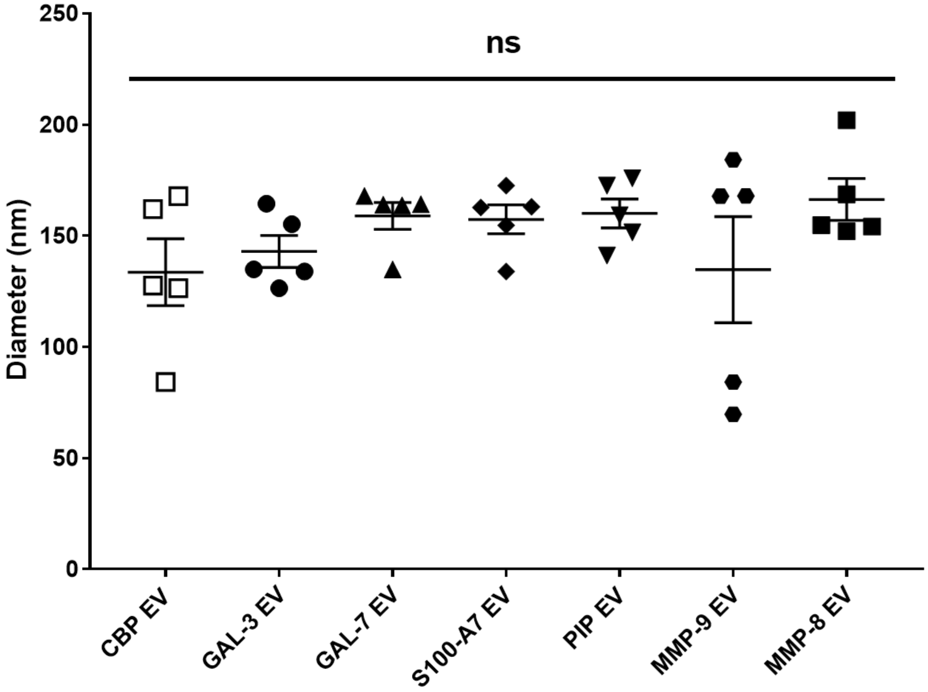

Figure S4. The size difference between CBP EVs and engineered EVs is not statistically significant. The CBP EV size measured by DLS was 127.59 nm ± 57.87. All genetically engineered EVs derived from H1ME-5 cells had a similar size of 145.61 nm ± 75.89 when measured by DLS (n = 5).

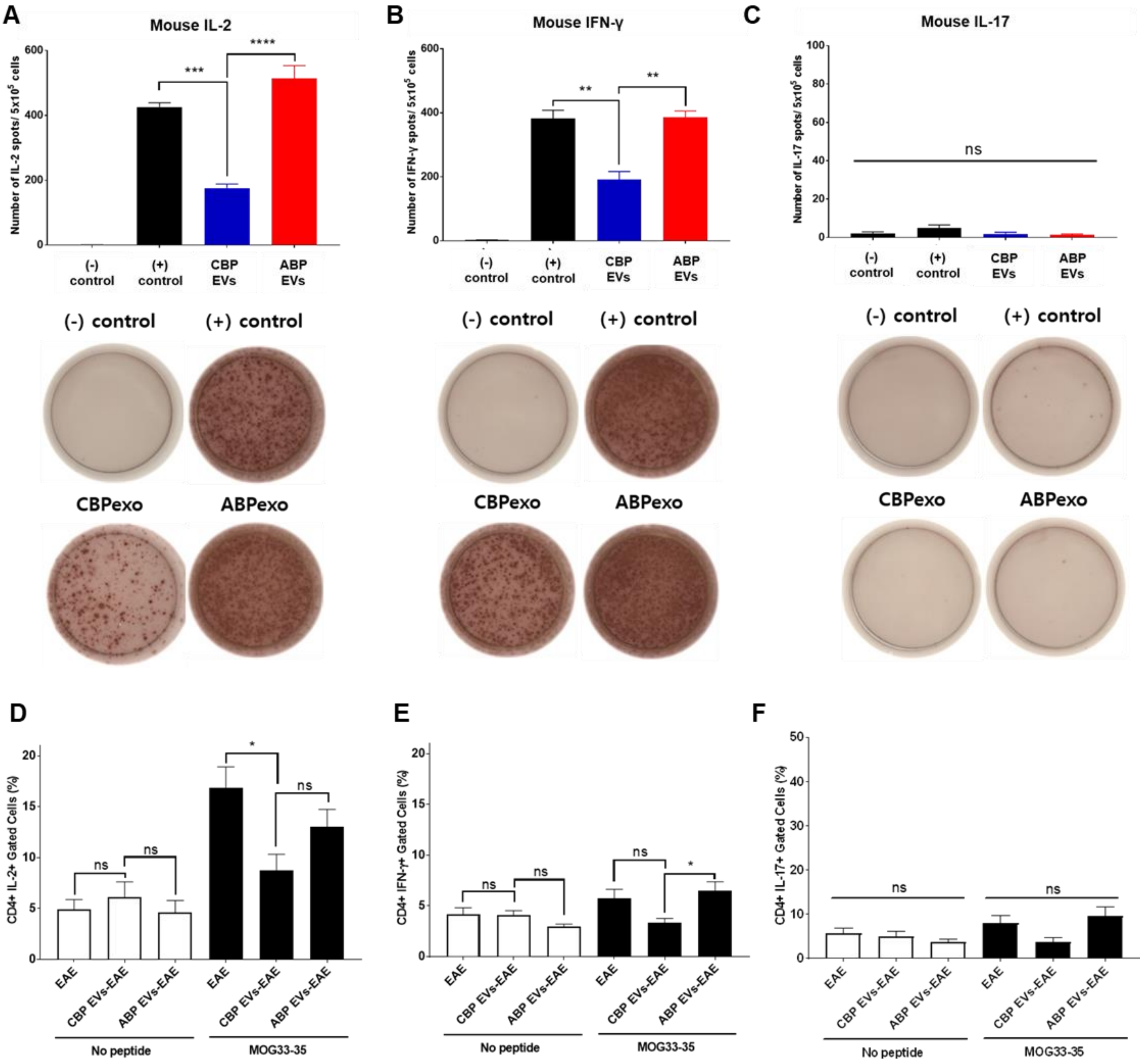

Figure S5. ELISPOT assay was performed to quantify the levels of (A) IL-2-, (B) IFN-γ-, and (C) IL-17-secreting T cells at 6 days. Mouse splenocytes were stimulated with CBP or ABP EVs, and the IL-2-, IFN-γ-, and IL-17-secreting cell patterns were observed. (D) IL-2, (E) IFN-γ, and (F) IL-17 cytokine-gated CD4+ T cells in the presence or absence of MOG33-55 peptide were analyzed for comparison with those of the EAE control, CBP EV-injected EAE, and ABP EV-injected EAE groups using intracellular cytokine staining (n = 4).
